# Progress towards measles elimination in Eritrea: 2003 - 2018

**DOI:** 10.11604/pamj.supp.2020.35.1.19126

**Published:** 2020-01-03

**Authors:** Tedros Yehdego, Tzeggai Kidanemaryam Yhdego, Balcha Masresha, Azmera Gebreslassie, Reggis Katsande, Daniel Fussum, Emmaculate Lebo, Josephine Namboze

**Affiliations:** 1 National Immunisation Program, Ministry of Health, Eritrea; 2 WHO Eritrea Country Office, Asmara, Eritrea; 3 WHO Regional Office for Africa, Brazzaville, Congo; 4 WHO Inter-country Team for Eastern and Southern Africa, Harare, Zimbabwe

**Keywords:** Measles elimination, Eritrea, immunisation, vaccine, Africa

## Abstract

**Introduction:**

The Expanded Program on Immunisation (EPI) has been operational in Eritrea since 1980. Eritrea has endorsed the resolution of the Regional Committee of the World Health Organisation African region, committing to a measles elimination goal for 2020 in the African Region. The country is implementing the recommended strategies.

**Methods:**

We reviewed administrative coverage and WHO UNICEF coverage estimates for Diphtheria-Pertussis-Tetanus (DPT) and measles routine vaccination, as well as for measles supplemental immunization activities. We reviewed national surveillance performance and analyzed the epidemiological trends of measles as reported in the case-based surveillance database.

**Results:**

Eritrea has maintained more than 90% coverage with the first dose of measles vaccine at national level since 2001 and 88% MCV2 coverage from 2015 - 2017 according to the WHO-UNICEF coverage estimates. Since 2011, the country has not met the surveillance performance target of at least 80% districts reporting suspected measles cases with blood specimen. Measles incidence was between 16.8 - 24.7 cases per million population in the period 2015 - 2018. The mean and median age of confirmed measles cases was more than 10 years in 8 of the 14 years covered by the analysis. In 2017, Eritrea reported 1,199 cases of measles which differs significantly from the 185 suspected cases in the case based surveillance database for the same year. Eritrea has maintained high coverage for MCV1 and MCV2 and made progress towards measles elimination. However, the country has gaps in surveillance performance which may mask the true incidence of measles.

**Conclusion:**

In order to attain elimination of measles, Eritrea needs to implement measures to improve surveillance quality, to conduct regular risk assessment and implement targeted measures to close immunity gaps. In addition, setting up a national committee for the verification of measles elimination will help the country document progress and also to highlight and advocate for addressing issues related to data quality and performance gaps.

## Introduction

Eritrea has a projected total population of 3,905,066 in 2018 including an estimated 117,152 surviving infants. Children less than 5 years of age are estimated to make up 15% of the total population. The country is divided into six administrative regions known as Zobas: Gash Barka, Anseba, Debub, Maekel, Debubawi Keih Bahri and Semanawi Keih Bahri Zobas (Zones), which in turn are divided into 58 subzobas (sub-zones) [1]. The 2018 report of the UN Inter-Agency Group for Child Mortality Estimation indicates that, in Eritrea, under-five mortality rate was reduced from 151 per 1,000 live births in 1990 to 43 per 1,000 live births in 2017. Infant mortality rate was also reduced from 93 per 1,000 live births in 1990 to 32 per 1,000 live births in 2017 [2]. The second national Health Sector Strategic Development Plan of Eritrea for 2017 - 2021 (HSSDP-II) further aims to reduce under-five and infant mortality rates to 32 and 25 per 1,000 live births respectively [3].

The Expanded Program on Immunisation (EPI) has been operational in Eritrea since 1980. Currently, the EPI program is housed as a unit within the Department of Public Health and is responsible to the director of family and community health division. EPI service delivery is integrated with other maternal and child health services and it is delivered as a package in all health facilities. By 2017, the ministry of health was providing healthcare service through 349 health facilities, in three-tier structure - namely primary care level, secondary care level and tertiary care level. A total of 295 (85%) health facilities provide routine immunization services 6 days per week in the country. In addition, immunization service is provided at 450 outreach sites across the country [1]. The HSSDP-II prioritizes, among others, the delivery of accessible and equitable immunization service for children below 5 years of age using the reaching every district approach [3]. As of 2019, the national EPI schedule includes antigens against 11 vaccine preventable diseases. These include a dose of BCG vaccine provided at birth, three doses of pentavalent vaccine (DPT/HiB/hepatitis B) and pneumococcal vaccine (PCV13) at 6, 10 and 14 weeks of age, four doses of oral polio vaccine (at birth, at 6, 10 and 14 weeks), Injectable Polio Vaccine (IPV) at 14 weeks of age, 2 doses of measles-rubella vaccine at 9 months and 18 months of age, and 2 doses of rotavirus vaccines at 6 and 10 weeks of life. Every woman of childbearing age (15 - 45 years) is expected to receive 5 doses of tetanus toxoid and diphtheria (Td) vaccine as per the WHO recommendations [4].

In 2011, the WHO African Region adopted a regional measles elimination goal for 2020, comprised of the following targets: 1) ≥ 95% coverage with the first dose of measles-containing vaccine (MCV1) at national and district levels; 2) ≥ 95% coverage in all districts during measles supplemental immunization activities (SIAs); 3) confirmed measles incidence < 1 per million population in all countries; 4) attaining high quality measles surveillance - to investigate ≥ 2 cases of non-measles febrile rash illness (NMFRI) per 100,000 population annually, and to obtain a blood specimen from ≥ 1 suspected measles case in ≥ 80% of districts annually [5]. The regional measles elimination goal is reflected as one of the objectives of the regional immunisation strategic plan 2014 - 2020 [6]. In line with the Regional goals, the Eritrean national comprehensive Multi-year plan for immunization (2017 - 2021) aims to achieve > 95% vaccination coverage with the first dose of measles-rubella vaccine (MR1) and 90% with the second dose of MR by 2021 [1]. This manuscript aims to describe the performance of Eritrea in the implementation of measles elimination strategies, the epidemiology of measles in the country and the overall progress towards measles elimination as at end of 2018.

## Methods

**Routine immunization:** the antigens provided to eligible persons as part of the routine immunization service are recorded and reported by health facilities to the sub-zobas, and the zobas, and onward to the National Immunization Programme. Sub-national and national coverage is calculated against the respective denominator targets and the national level coverage is reported annually to WHO and UNICEF. WHO and UNICEF use coverage data from administrative reporting and from surveys to generate coverage estimates for each antigen provided through the routine immunization services [7]. We analyzed the administrative measles vaccination service data, coverage information from surveys and the annual WHO-UNICEF measles vaccination coverage estimates for Eritrea for the years 1993 - 2017.

**Supplemental immunization**: Eritrea has been conducting preventive measles supplemental immunization activities (SIAs) periodically since 2003. At the end of each SIAs, technical reports are compiled, and often coverage surveys are done to corroborate administrative coverage levels. We reviewed the various technical reports and coverage survey results following the measles SIAs conducted in Eritrea between 2003 and 2018 [8].

**Measles surveillance and disease incidence:** Eritrea established measles case-based surveillance, with the support of a national serological laboratory for the confirmation of measles cases starting in 2005. Measles surveillance protocols as well as the methods and tools used by the measles serological laboratory network are standardized across the WHO African Region [9]. We analyzed the surveillance database for the years 2005 to 2018. We reviewed the epidemiological pattern of measles cases confirmed by laboratory testing, epidemiological linkage or clinical criteria. Measles IgM negative specimens are tested for rubella IgM as part of the standard protocol. We reviewed the number of lab confirmed rubella cases reported in the same period.

Measles surveillance performance is monitored using standard performance indicators. The two principal performance indicators are: non-measles febrile rash illness rate (target of at least 2 per 100,000 population) and the proportion of districts that have investigated at least one suspected case of measles with blood specimen per year (target at least 80% of districts per year). The incidence of confirmed measles is calculated as a rate per million, by dividing the total number of confirmed measles cases (confirmed by laboratory, epidemiological linkage and clinical criteria) by the total population [9]. In addition to the analysis of data from the case-based measles surveillance database, we reviewed the official number of measles cases reported by the country annually to WHO and UNICEF through the Joint Reporting Form (JRF) [10].

## Results

Routine immunization: national coverage with the first and third doses of Diphtheria-Pertussis-Tetanus containing vaccine (DPT1 and DPT3) sharply increased from 49% and 32% respectively in 1993 to 97% and 93% in 1999 according to the WHO UNICEF coverage estimates. During the same period, the first dose of measles vaccine (MCV1) coverage improved from 34% to 88%. Coverage for all antigens dropped by about 10 percentage points in 2000 but recovered by 2002. From 2003 until 2017, vaccination coverage with the primary antigens has been maintained at above 90% (Table 1).

**Table 1:** DPT1, DPT3 and measles first and second dose vaccination coverage according to the WHO UNICEF coverage estimates, Eritrea, 1993 - 2017

	DPT1	DPT3	MCV1	MCV2
1993	49%	32%	34%	
1994	61%	49%	51%	
1995	68%	58%	58%	
1996	75%	66%	66%	
1997	83%	75%	73%	
1998	90%	86%	79%	
1999	97%	93%	88%	
2000	88%	81%	76%	
2001	91%	86%	84%	
2002	94%	90%	83%	
2003	97%	93%	92%	
2004	99%	98%	96%	
2005	99%	96%	95%	
2006	97%	94%	95%	
2007	98%	95%	96%	
2008	98%	95%	96%	
2009	98%	95%	97%	
2010	98%	95%	97%	
2011	97%	94%	98%	
2012	97%	94%	98%	
2013	97%	94%	94%	
2014	97%	94%	90%	
2015	98%	95%	97%	88%
2016	97%	95%	99%	88%
2017	97%	95%	99%	88%

**Figure 1:**
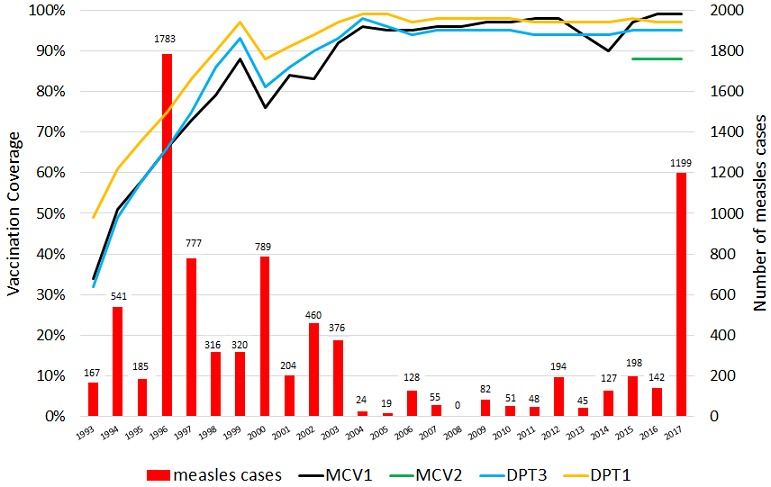
Officially reported measles cases, and WHO UNICEF coverage estimates. Eritrea. 1993 - 2017.

Eritrea introduced the second dose of measles vaccine (MCV2) in the routine immunization schedule in July 2012, providing it to children starting at 18 months of age. However, the country started reporting MCV2 coverage to the WHO and UNICEF in 2015. The WHO-UNICEF estimates of MCV2 coverage for 2015 - 2017 have been consistently 88%, with the drop-out rate between the first and second doses of measles vaccine staying at 9 - 11% (Table 1, Figure 1). Eritrea introduced rubella vaccine into the routine immunization schedule in December 2018, following a nationwide measles -rubella catch-up SIAs.

The Demographic Health Survey (DHS) done in Eritrea in 2002 indicated BCG coverage of 91.4%, DPT3 coverage of 82.8% and MCV1 coverage of 84.2% [11]. The National EPI coverage survey done in 2017 indicated that 98.9% had received BCG, while 97.3% received the third dose of pentavalent vaccine and 96.8% were vaccinated with the first dose of measles vaccine. Coverage with the second dose of measles vaccine (MCV2) was 86.7% among 24 - 35 months old children. According to the 2017 survey, MCV1 coverage by province ranged from 92.1% in Gash Barka to 99.2% in Maekel and MCV2 coverage ranged between 66.5% in Debubawi Keih Bahri and 92.7% in Maekel [12].

**SIAs:** Eritrea conducted the first preventive SIAs against measles in 2003, targeting children from 9 months to 14 years of age and reaching a total of 1,047,862 children (82% of the target). In subsequent years, the country conducted measles follow-up SIAs every 3 years and a wide age range measles-rubella (MR) catch-up SIAs in 2018. Administrative coverage at national level was less than 85% in all SIAs except in 2006. However, coverage surveys conducted after the 2012 and 2018 SIAs both indicated coverage of more than 95% at national level (Table 2) [8, 13]. During the MR catch-up SIAs of 2018, the administrative coverage ranged from 73% in Anseba to 95% in Gash Barka (Table 3). Post-campaign survey results showed > 95% coverage in all Zobas. Only 5.4% children aged 9 - 59 months of age had no prior measles vaccination prior to the MR SIAs in 2018, according to the survey report.

**Table 2:** Measles supplemental immunization activities (SIAs) coverage in Eritrea, 2003 - 2018

Year	Age group of children targeted	Number of children vaccinated	Administrative coverage at national level (% of target)	% districts with administrative coverage ≥ 95%	Coverage survey result
2003	9 months - 14 years	1,047,862	82%		Not Done
2006	6 - 59 months	387,479	95%		Not Done
2009	9 - 59 months	285,285	82%		Not Done
2012	6 - 47 months	277,928	75%	16.0%	95.6%
2015	9 - 59 months	350,765	80.1%	36.2%	Not Done
2018	9 months - 14 years	1,276,364	84%	0.0%	97.8%

**Table 3:** Measles-rubella supplemental immunization activities (SIAs) administrative and survey coverage at Zoba level, Eritrea, 2018

Zoba	Target Population	Vaccinated children	Administrative coverage	Survey coverage
Maekel	223,020	179,046	80.30%	99.40%
Gash Barka	396,981	376,699	94.90%	97.80%
Anseba	224,056	163,163	72.80%	99.60%
Debub	368,055	301,595	81.90%	95.30%
Semenawi Keih Bahri	191,306	154,047	80.50%	99.70%
Debubawi Keih Bahri	36,070	32,200	89.30%	99.60%
National	1,439,488	1,206,750	83.80%	97.80%

**Measles and rubella surveillance performance:** the national level target of 2 non-measles febrile rash illness cases per 100,000 population (NMFRI) has been met since 2010 but the target was missed in 2007 - 2009. However, since 2011, Eritrea has not attained 80% target for districts reporting suspected measles cases with blood specimen (Table 4). The national serological laboratory for the confirmation of suspected measles and rubella cases has recently been accredited since 2010, though with varied performance. Training was done in October 2018. There is no sentinel surveillance system in place to investigate and report congenital rubella syndrome (CRS) cases and no retrospective review has been done to date.

**Table 4:** Measles case-based surveillance performance, Eritrea, 2005 - 2018 (Source: Measles case-based surveillance system)

Year	Total suspected measles cases	Suspected measles cases that were investigated	Non-measles febrile rash illness (NMFRI) rate – [Target: ≥ 2 cases per 100,000 population]	% districts investigating suspected cases per year [Target: ≥ 80% districts]
2005	159	155	4.2	100%
2006	125	125	3.9	100%
2007	52	52	1.6	100%
2008	61	59	1.8	100%
2009	45	45	1.2	30%
2010	168	168	4.6	83%
2011	145	145	3.6	75%
2012	243	243	4	66%
2013	139	139	2.3	41%
2014	103	102	2.6	36%
2015	195	195	2.9	59%
2016	334	334	6.8	60%
2017	185	183	3.4	36%
2018	155	154	2.1	53%

**Figure 2:**
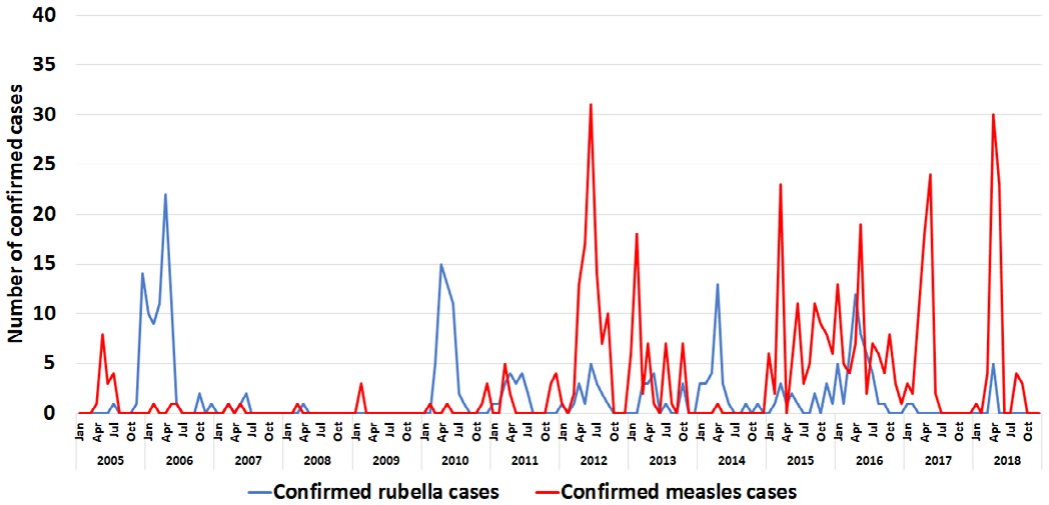
Monthly trends of reporting of confirmed measles and rubella in Eritrea. 2005 - 2018.

**Measles and rubella incidence:** between 2005 and 2018, Eritrea reported a total of 2,112 suspected measles cases through the case-based surveillance system, of which 529 were confirmed by laboratory, epidemiological linkage or clinical compatibility. On average, annually, 150 suspected measles cases were reported through the case-based surveillance system. There were 278 laboratory confirmed rubella cases in the same period (Table 5). The incidence of confirmed measles in Eritrea ranged from 0.3 per million in 2008 and 2014 to 24.7 per million population in 2015. The country reported measles incidence levels of less than 1 measles case per million in 2007, 2008, 2009 and 2014. The incidence of confirmed measles was more than 10 per million in 7 out of the 14 years analyzed, including from 2015 - 2018. The peak period of measles occurrence in Eritrea is between January and May in most years. Similar peaks are seen in the occurrence of lab confirmed rubella cases in the first half of the year (Figure 2).

**Table 5:** Measles incidence and age patterns by year 2005 – 2018, Eritrea (Source: Measles case-based surveillance system)

Year	Number of suspected measles cases	Number of confirmed rubella cases	Number of confirmed measles cases	Incidence of confirmed measles per 1,000,000 population	Mean age (in years) of confirmed measles cases	Median age (in years) of confirmed measles cases	% of measles cases unvaccinated
2005	159	16	32	10.6	13	7	100%
2006	125	67	9	3	7	6	0%
2007	52	4	2	0.6	4.5	4.5	0%
2008	61	1	1	0.3	0	0	0%
2009	45	0	3	0.9	22.3	25	100%
2010	168	47	6	1.7	18.5	22	75%
2011	145	18	14	3.9	18.9	18	100%
2012	244	17	95	24	23.8	25	88%
2013	139	15	50	13.1	23.6	21	91%
2014	105	32	1	0.3	3	3	0%
2015	195	14	104	24.7	25.5	27	89%
2016	334	44	81	16.8	20.9	22	81%
2017	185	2	62	16.8	10.7	9	51%
2018	155	5	69	17.7	13.7	12	63%

More than half (60.5%) of the confirmed measles cases reported between 2005 and 2018 are more than 15 years of age. This high proportion was also evident from 2015 - 2018, with children more than 15 years of age comprising of more than 40% of all confirmed measles cases in 2015, 2016 and 2018. Both the mean and median age of confirmed measles cases were more than 10 years in 8 of the 14 years covered by our analysis. All of the years with documented incidence of more than 1 per million (except 2006) had mean age of measles cases of more than 10 years.

Vaccination status was not documented in the records of 162 of the 529 confirmed measles cases. Of the remaining confirmed cases whose status was documented, the proportion of cases with no history of measles vaccination ranges between 51% and 89% in the years 2015 - 2018 (Table 5). The comparison of the number of confirmed measles cases in the case-based surveillance database and in the official annual country report to WHO and UNICEF through the Joint Reporting Form (JRF) showed differences. In 2006, 2007 and 2015, the officially reported figure is closer to the number of suspected cases in the case-based surveillance database. However, the comparison shows significant difference in the other years. Especially in 2017, a total of 1,199 measles cases were officially reported to WHO and UNICEF, while the case based surveillance database contained 185 suspected cases of which only 65 were confirmed. There is no detailed epidemiological data or investigation report available to explain this spike in 2017 (Table 6). Eritrea does not yet have any documented measles or rubella viral strains.

**Table 6:** Comparison of the reported measles cases through the case-based surveillance system and the aggregate annual official reporting to WHO and UNICEF through the joint reporting form 2005 - 2018

Year	Measles case reports		
	Total suspected measles cases in the national measles case-based surveillance database	Confirmed measles cases in the national measles case-based surveillance database	Measles cases reported officially to WHO and UNICEF through the JRF
2005	159	32	19
2006	125	9	128
2007	52	2	55
2008	61	1	0
2009	45	3	82
2010	168	6	51
2011	145	14	48
2012	243	95	194
2013	139	50	45
2014	103	1	127
2015	195	104	198
2016	334	81	142
2017	185	62	1199
2018	155	69	70

## Discussion

Eritrea has made significant progress towards measles elimination. The country has managed to sustain very high coverage with MCV1 for more than 10 years and an equally high coverage with the second dose of measles vaccine since it started reporting MCV2 coverage. The dropout rate between these two doses is less than 11% at national level in the three years of reporting. However, coverage is not homogeneous across all Zobas especially for MCV2 coverage. In order to achieve and sustain measles elimination, Eritrea will need to reach at least 95% coverage with both MCV1 and MCV2.

The fact that Eritrea has had low routine immunization and SIAs administrative coverage, but significantly high coverage by surveys indicates that the official population figures may be overestimated. These denominator figures are generated as projections. Eritrea has never done any census. The wide age-range MR SIAs of 2018 has attained ≥ 95% coverage by survey across all provinces, and it will likely take care of any immunity gaps among the targeted population of children 9 months to 15 years of age. The expected impact of the MR SIAs on measles and rubella incidence among the targeted cohort of children under 15 years of age will need to be documented through a sensitive surveillance system [14].

The total number of measles cases reported has markedly declined following the SIAs in 2003. Incidence of confirmed measles has been low until 2012. In the years 2013 - 2018, measles incidence rate was between 10 and 20 per million population despite the high coverage attained for many years. The country has had a large proportion of school age children and adults among measles cases in the past decade, with mean age of confirmed measles being higher than 10 years of age. It is evident that measles incidence is driven by susceptible in the adolescent and adult age group. This epidemiological shift to older age groups can be explained on the low population density and the relatively high measles vaccination coverage over the last two decades [15].

Measles surveillance is integrated with active surveillance for acute flaccid paralysis in Eritrea. However, there are gaps in case-based surveillance performance for measles, with the country failing to attain the target for district reporting since 2011. Information on the vaccination status of cases was missing in 30% of the 529 confirmed cases from 2005 - 2018. These performance gaps will need to be addressed through further investigation to identify the specific non-reporting districts and the factors leading to the weak performance. In addition, there is a need to investigate and document all outbreaks of measles in order to identify the specific populations that may be at risk and take the necessary measures. This will help to better understand the epidemiological factors and populations at risk in Eritrea.

With the gaps documented in surveillance performance, the information on incidence from the surveillance system will need to be interpreted cautiously. The analysis has shown that the surveillance system was not able to launch a detailed investigation and documentation on the febrile rash illness cases which occurred in 2017 as reported to WHO through the joint reporting format. In addition, the discrepancy in the number of measles cases reported in various years through the case-based surveillance system and the annual summary reports to WHO and UNICEF indicates the need for regular harmonization of data, and for aggressive efforts to investigate all suspected cases and take timely programmatic action to limit measles outbreaks spread as much as possible.

The national EPI policy emphasizes that immunization service provision will be done as an integral part of the primary health care services including prevention and control of childhood diseases, growth monitoring, information, education and communication, nutritional advice, ante-natal, post-natal care and family planning. It also states that provision of insecticide treated bednets, vitamin A supplementation and de-worming shall be supplied through routine immunization and campaign settings, with a view to reduce missed opportunities. Eritrea is eligible for Global Alliance for Vaccines and Immunization (GAVI) support. Starting from 2016 the government has started co-financing 20% of the total costs on traditional vaccines, including measles vaccine [4].

The sustained high immunization coverage in Eritrea is a function of the strength of the immunization program. A national immunization program review done in 2016 identified programmatic strengths that included the delivery of integrated services offered 6 days a week, very good caretaker awareness of the benefits of immunization and high demand for services. However, it was also noted that lack of transportation may pose a risk of delaying vaccine availability to the population, and to reaching out to hard to reach populations [16, 17]. The country has conducted very good programmatic preparation and roll out of MCV2 which has also contributed very well to the sustained high coverage of MCV2 [17, 18]. Following the introduction of MCV2, an evaluation done in April 2015 found out that staff knowledge was satisfactory, monitoring of service data was being done systematically, cold chain capacity was adequate, supervisory support to the health facility level was being provided regularly and that the community awareness of the vaccine schedule was good. However, the evaluation identified the need for more active monitoring of adverse events following immunisation [18].

It has been documented that having a cadre of community health workers, immunization services tailored to community needs, health system and community partnership, and a regular review of health worker performance are key drivers of improvement in district level immunization program coverage in the African context [19]. The HSSDP II plans to expand and improve the work of community health workers guided by an integrated comprehensive community strategy. In the area of maternal and child health, there are plans to scale up community involvement on microplanning at district level to address population groups in less accessible geographical areas [3]. These activities will have a strong impact on the progress towards sustainable measles elimination in Eritrea.

The World Health Organisation has developed a framework for the verification of measles elimination which has set the criteria for defining measles elimination and the processes for verifying measles elimination in a country. The framework requires that countries establish the necessary independent structures responsible for compiling the programmatic and epidemiological information necessary to assess progress and document, measles elimination [20]. This includes the establishment of National Verification Committees (NVC) with the primary responsibility for guiding countries in the preparation of their documentation of progress towards the achieve¬ment of measles elimination and the Regional Verification Commission (RVC), which validates and verifies elimination in each country and eventually in the Region [21]. As of April 2019, Eritrea has not yet established an NVC or started the documentation of progress towards measles elimination.

**Limitations:** this study has limitations. First, there may be inaccuracies in administrative coverage monitoring. Surveys have been shown to provide higher coverage than reported data due to inaccuracies of denominators used for coverage monitoring. Secondly, the study did not look at the measles laboratory performance indicators or the quality of serological specimens. Thirdly, the weaknesses in surveillance performance and the gaps in the investigation of cases and outbreaks may conceal the true incidence and epidemiological pattern of measles in the country.

## Conclusion

In order to further advance towards the measles elimination goal, we recommend that Eritrea strengthen its surveillance system, investigate and fully document outbreaks of measles, ensure that all districts report and investigate suspected cases, conduct regular risk assessment to identify and address immunity gaps. The triangulation of data from coverage monitoring, surveillance and risk assessment exercises helps to target tailored interventions within the routine immunization service delivery platform or through the Periodic Intensification of Routine Immunization (PIRI) model [22]. With the current levels of coverage in the childhood population, the country can extend its inter-SIAs interval to 4 - 5 years, without risking a rapid accumulation of young children susceptible to measles infection. Depending on the findings from risk assessment exercises and disease surveillance, SIAs may be tailored to target susceptible adolescent and adult populations in specific areas, in addition to young cohorts. Eritrea should set up a national verification committee to document the progress with measles elimination. This will serve as an opportunity to raise the profile of measles elimination in the national health agenda, and to advocate for rubella/congenital rubella syndrome (CRS) control in the country. As a way of monitoring the impact of the introduction of rubella vaccine, the country should consider doing a retrospective review of CRS in a few tertiary care centers and initiate sentinel CRS surveillance on a prospective basis.

### What is known about this topic

- Eritrea has managed to reduce under five and infant mortality significantly in the past 25 years;
- Eritrea has been implementing measles elimination strategies since 2003;
- Eritrea introduced measles vaccine MCV2 in 2012.

### What this study adds

- Eritrea has maintained high MCV1 and MCV2 coverage, with drop-out rates of less than 11% between the two doses;
- Measles surveillance performance gaps persist, and there is a discrepancy in the number of reported measles cases between the case-based surveillance data and aggregate reporting;
- The mean and median age of measles cases in Eritrea has been mostly above 10 years in the past 15 years.

## Competing interests

The authors declare no competing interests.
